# Supplementary material for: Robust real-time imaging through flexible multimode fibers
Source: Sci Rep. 2023 Jul 14;13:11371. doi: 10.1038/s41598-023-38480-4 (PMC10349048; doi:10.1038/s41598-023-38480-4)
Supplement: Supplementary file 1 — Supplementary Information 1. [file 41598_2023_38480_MOESM1_ESM.pdf]

# Robust real-time imaging through flexible multimode fibers: Supplementary Material

**Abdullah Abdulaziz[1,*], Simon Peter Mekhail[2], Yoann Altmann[1], Miles J. Padgett[2], and Stephen McLaughlin[1]**

[1]Heriot-Watt University, School of Engineering and Physical Sciences, Edinburgh, EH14 4AS, United Kingdom
[2]University of Glasgow, School of Physics and Astronomy, Glasgow, G12 8QQ, United Kingdom
[*]a.abdulaziz@hw.ac.uk

## Supplementary Note 1: The experimental geometry

We use a Q-switched, 21.7 kHz repetition rate, 532 nm wavelength, 700 ps, pulsed laser (Teem Photonics SNG-100P-1x0) for all imaging procedures in this work. The beam is expanded such that the $1/e^2$ diameter fits inside the short axis of a DMD (Vialux V-7000). A constant grating is applied to the DMD to direct diffracted light away from the $0^{\text{th}}$ order where phase shaping may be applied. The beam is then directed through a half wave plate (HWP) which is used to control the power incident on the fiber and direct a small portion of the light to a triggering photodiode (PD) through a polarizing beam splitter (PBS) as shown in Supplementary Figure 1. The light is then converted into circularly polarized light with a quarter wave plate (QWP). In a plane Fourier to the DMD, the light is spatially filtered to select the $1^{\text{st}}$ order of the diffraction pattern. The beam is then collimated with an objective lens (Olympus 40x Plan-N) and the plane wave incident on the fiber is coupled into the fiber core. This makes the fiber input facet conjugate to the plane of the DMD. For brevity, extra relay lenses are excluded from Supplementary Figure 1, however the magnification from the fiber to the DMD is set at 1600:9. The illumination fiber is a 50 cm, graded index, 0.275 NA, 62.5 $\mu$m core diameter, fiber (Thorlabs GIF625). It is epoxied into a 20 G, 3 cm, needle alongside a 50 cm, step index, 0.39 NA, 400 $\mu$m core diameter, collection fiber (Thorlabs FT400UMT). Both fibers are housed in a common jacket and are therefore both subject to bending by the bending arm and rotation stage.

During calibration, an internal reference beam is used to measure the phase of the probed fiber modes. The reference beam is made by randomly selecting 26 points at a plane Fourier to the DMD and exciting these points using a super position of grating frequencies on the DMD. A probe mode is then added to the super position and phase stepped through the reference. The resulting interference patterns are incident on a white screen and are recorded by a camera (Hamamatsu Orca Flash 4.0). From these images, the complex transmission matrix of the fiber can be determined, allowing for the calculation of the optimal DMD phase mask needed to generate any spot within the fiber's illumination cone.

For imaging, the camera is not used. Rather, an object is placed at the screen and the calculated DMD phase masks are displayed on the DMD at the rate of one pattern per laser pulse. The trigger PD starts a set number of samples being recorded by the APD (Menlo Systems APD210) at 2.5 GS/s as the pulse propagates to the object and back. The reflectivity of the object is given by the peak intensity of the return pulse and, as such, the maximum of the return signal is recorded giving a single value for each scanned spot. This time gating increases the signal to noise ratio of the system. As the system is numerically trained and physically tested, great care was taken to ensure that the perceived resolution of the images being recorded by the fiber was identical to that used in the training.

Calibration was performed once in this work at an extremum of the fiber bend apparatus and, as such, most of the imaging was performed with unknown speckle patterns. Reconstructing such images with unknown and unoptimized speckle patterns was the task borne out by the GMVAE. To ensure the expected speckle patterns differed significantly from the calibrated configuration, the bend arm and the rotation stage worked in opposition to one another as seen in Supplementary Figure 1, that is, while one pulls the fiber in one direction the other pushes another part of the fiber in the opposing direction. The bend arm had a range of movement of 8 cm, and the rotation stage turned the needle, camera, and screen together through 50°. At each of the 11 stops, the speckle patterns were recorded using the same DMD phase masks calculated during the calibration in the case of the calibrated experiment, and the same randomly selected DMD phase masks in the uncalibrated experiment. A selection of these were then used in simulating the training data for the GMVAE.

## Supplementary Note 2: Gaussian mixture variational autoencoder

Recall that our aim is to learn a configuration-agnostic variational autoencoder that can estimate the underlying object $\boldsymbol{x}$ and its class from APD measurements $\boldsymbol{y}$ corresponding to a bend that was not part of the training set. The class of each image can be either represented by a scalar $c$ or a one-hot encoded vector $\boldsymbol{c}$ of length $k$, i.e., the number of classes. In GMVAE, the image to

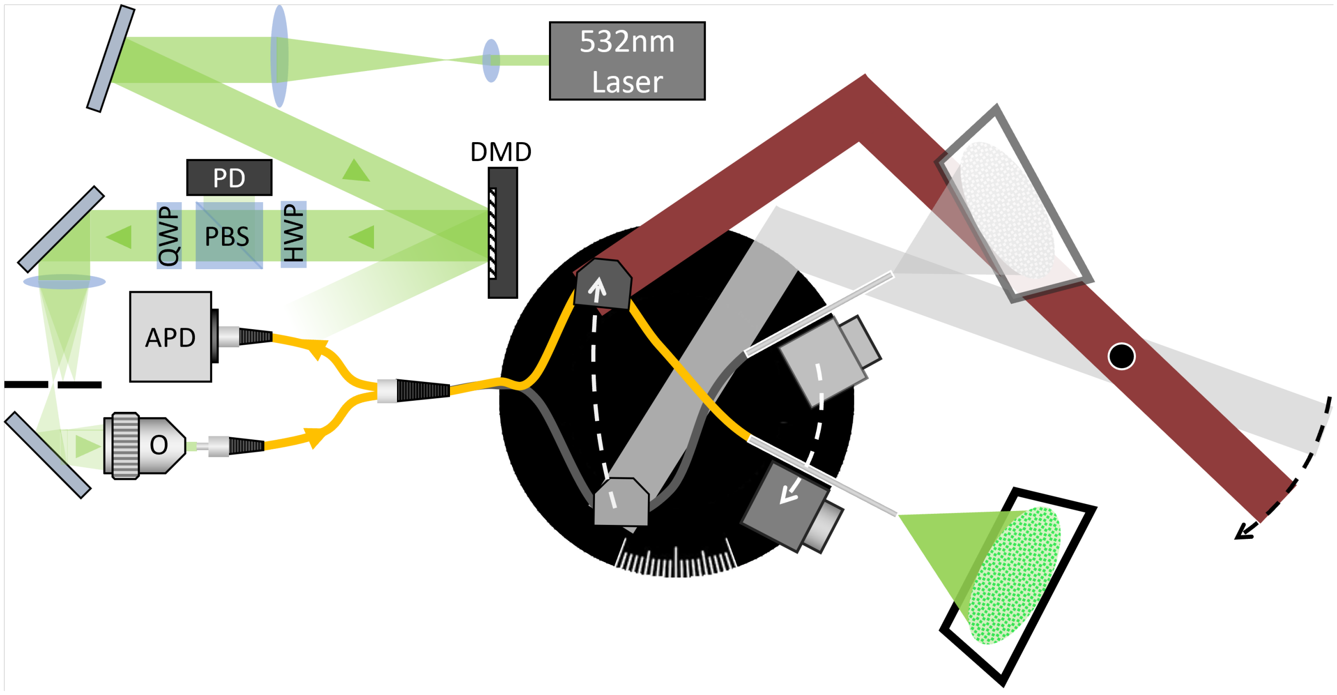


**Supplementary Figure 1.** Schematic illustrating the calibration, imaging, and bending apparatus used in this work. The grayed-out camera, screen, needle, and bend arm represent the fiber configuration at the starting position where calibration is performed, while the corresponding fully saturated portion depicts the fiber configuration at the final position. **DMD**: digital micromirror device; **HWP**: half-wave plate; **PBS**: polarizing beam splitter; **QWP**: quarter-wave plate; **PD**: photodiode; **O**: objective lens; **APD**: avalanche photodiode.

be reconstructed $\boldsymbol{x}$ is assumed to follow a generative process:

$$\mathrm{p}_{\boldsymbol{\theta}}(\boldsymbol{x}, z, \boldsymbol{c}) = \mathrm{p}_{\boldsymbol{\theta}}(\boldsymbol{x}|z)\ \mathrm{p}_{\boldsymbol{\theta}}(z|\boldsymbol{c})\ \mathrm{p}(\boldsymbol{c}), \tag{1a}$$

$$c \sim \mathrm{Cat}(c|\ 1/k), \tag{1b}$$

$$z|\boldsymbol{c} \sim \mathcal{N}(z|\ \boldsymbol{\mu}_{z_{\boldsymbol{\theta}}}(\boldsymbol{c}), \boldsymbol{\sigma}^2_{z_{\boldsymbol{\theta}}}(\boldsymbol{c})), \tag{1c}$$

$$\boldsymbol{x}|z \sim \mathcal{N}(\boldsymbol{x}|\ \boldsymbol{\mu}_{\boldsymbol{x}_{\boldsymbol{\theta}}}(z), \boldsymbol{\sigma}^2_{\boldsymbol{x}_{\boldsymbol{\theta}}}(z)), \tag{1d}$$

where $z \in \mathbb{R}^d$ is the latent variable. The vectors $\boldsymbol{\mu}_{z_{\boldsymbol{\theta}}}$, $\boldsymbol{\sigma}^2_{z_{\boldsymbol{\theta}}}$, $\boldsymbol{\mu}_{\boldsymbol{x}_{\boldsymbol{\theta}}}$ and $\boldsymbol{\sigma}^2_{\boldsymbol{x}_{\boldsymbol{\theta}}}$ are given by the GMVAE generative network with parameters $\boldsymbol{\theta}$. The objective of GMVAE is to approximate the posterior distribution $\mathrm{p}(z, \boldsymbol{c}|\boldsymbol{y})$ which is intractable and typically approximated by a factorized posterior, namely the inference model:

$$\mathrm{q}_{\boldsymbol{\phi}}(z, \boldsymbol{c}|\boldsymbol{y}) = \mathrm{q}_{\boldsymbol{\phi}}(z|\boldsymbol{y}, \boldsymbol{c})\ \mathrm{q}_{\boldsymbol{\phi}}(\boldsymbol{c}|\boldsymbol{y}), \tag{2a}$$

$$c|\boldsymbol{y} \sim \mathrm{Cat}(c|\ \pi_{\boldsymbol{\phi}}(\boldsymbol{y})), \tag{2b}$$

$$z|\boldsymbol{y}, \boldsymbol{c} \sim \mathcal{N}(z|\ \boldsymbol{\mu}_{z_{\boldsymbol{\phi}}}(\boldsymbol{y}, \boldsymbol{c}), \boldsymbol{\sigma}^2_{z_{\boldsymbol{\phi}}}(\boldsymbol{y}, \boldsymbol{c})), \tag{2c}$$

where $\boldsymbol{\phi}$ are the parameters of the inference network. The categorical distribution $\mathrm{Cat}(c|\ \pi_{\boldsymbol{\phi}}(\boldsymbol{y}))$ is approximated using the Gumbel-Softmax (Concrete) distribution[1,2]. The parameters of the inference and generative networks, $\boldsymbol{\phi}$ and $\boldsymbol{\theta}$, are learnt by maximizing the evidence lower-bound (ELBO)[3,4], that is a lower bound on the log probability of the observations:

$$\mathrm{ELBO} = -\alpha \mathrm{KL}(\mathrm{q}_{\boldsymbol{\phi}}(z|\boldsymbol{y}, \boldsymbol{c}) \| \ \mathrm{p}_{\boldsymbol{\theta}}(z|\boldsymbol{c})) - \beta \mathrm{KL}(\mathrm{q}_{\boldsymbol{\phi}}(\boldsymbol{c}|\boldsymbol{y}) \| \ \mathrm{p}(\boldsymbol{c})) + \omega \mathrm{E}_{\mathrm{q}_{\boldsymbol{\phi}}(z|\boldsymbol{y}, \boldsymbol{c})}[\log \mathrm{p}_{\boldsymbol{\theta}}(\boldsymbol{x}|z)], \tag{3}$$

where $\alpha, \beta, \omega > 0$ are hyper-parameters controlling the weights of the different priors. KL stands for the Kullback-Leibler divergence and the last term is given by the reconstruction loss.

The classification accuracy can be greatly enhanced by learning an appropriate distance metric from the labels. Therefore, we adopt the triplet embedding loss as an auxiliary loss function to regularize the latent space[5,6]. The triplet loss penalizes large

distances between features belonging to the same class while penalizing small distances between features with non-matching labels. The final objective for the adopted GMVAE takes the form:

$$\mathscr{L}_{\text{GMVAE}} = \text{ELBO} + \gamma \mathscr{L}_{\text{triplet}}, \tag{4}$$

where $\mathscr{L}_{\text{triplet}}$ is the triplet embedding loss and $\gamma > 0$ is the hyper-parameter controlling the weight of the auxiliary loss function. Using grid search, we found that $\alpha = 1,\ \beta = 200,\ \omega = 50$ and $\gamma = 50$ gives optimal results. The same parameters are fixed for all experiments.

## Supplementary Note 3: GMVAE architecture

The GMVAE architecture consists of inference and generative networks. The inference network starts with an encoder to learn the features of the input measurement vector $\boldsymbol{y}$, dubbed $\hat{\boldsymbol{y}}$. The encoder consists of three convolutional layers each followed by a batch normalization layer and a rectified linear unit (ReLU) as an activation function. The classification network $\text{q}_{\boldsymbol{\phi}}(\boldsymbol{c}|\boldsymbol{y})$ consists of two dense layers of size 1024 and $k$ (here $k = 8$ classes) and a dropout layer of rate 0.2 between the dense layers to avoid over-fitting. The network input is the learnt features $\hat{\boldsymbol{y}}$ and the output is the sampled categories given by the Gumbel-Softmax (Concrete) distribution[1,2]. The inference network $\text{q}_{\boldsymbol{\phi}}(\boldsymbol{z}|\boldsymbol{y},\boldsymbol{c})$ has two dense layers of size $d = 1024$ to produce the latent space mean $\boldsymbol{\mu}_{\boldsymbol{z_\phi}}$ and variance $\boldsymbol{\sigma}^2_{\boldsymbol{z_\phi}}$. The inference network input is the concatenation of $\hat{\boldsymbol{y}}$ and the sampled categories and the output is the sampled latent space given by the reparameterization trick. Note that a dropout of rate 0.2 is applied on the features $\hat{\boldsymbol{y}}$ before concatenation with the categories to avoid features over-fitting. The prior network $\text{p}_{\boldsymbol{\theta}}(\boldsymbol{z}|\boldsymbol{c})$ is represented by two dense layers of size $d = 1024$ to produce the prior mean $\boldsymbol{\mu}_{\boldsymbol{z_\theta}}$ and variance $\boldsymbol{\sigma}^2_{\boldsymbol{z_\theta}}$, respectively. Finally, the generative network $\text{p}_{\boldsymbol{\theta}}(\boldsymbol{x}|\boldsymbol{z})$ consists of a dense layer of size 4096 followed by three transposed convolutional layers (each followed by a batch normalization layer and a ReLU activation function) and finally a dense layer of size $N = 4096$ with a logistic (sigmoid) activation function to produce the output vector $\boldsymbol{\mu}_{\boldsymbol{x_\theta}}$. GMVAE was trained over 300 epochs using the Adam optimizer[7] with a learning rate $10^{-4}$ and a batch size 100.

## Supplementary Note 4: AE and C-AE architecture

We recall that the performance of the GMVAE is compared to that of the autoencoder (AE)[8,9]. The structure of the AE follows the encoder–decoder architecture where the input measurements of size $64 \times 64$ go through the encoder which consists of three dense blocks connected by max pooling layers for downsampling. Each dense block contains multiple convolutional layers, each followed by a batch normalization layer and a ReLU activation function. Next, the downsampled map of the measurements goes through the decoder that consists of three dense blocks connected by upsampling layers. Similarly to[8,10] and as opposed to[9], we do not use fully-connected layers in the bottleneck of the AE as we notice that this leads to degradation in the reconstruction quality.

The classification accuracy of GMVAE is compared to that of the C-AE classifier. C-AE is trained on reconstructed images from AE and can only be trained after AE is trained while GMVAE is trained on measurements for the classification and reconstruction tasks simultaneously. C-AE has the same architecture as the classifier used in our GMVAE. It consists of three convolutional layers (each followed by a batch normalization layer and a ReLU activation function) followed by two dense layers of size 1024 and eight (number of classes) with a dropout layer of rate 0.2 between the dense layers to avoid over-fitting.

AE and C-AE were trained over 100 epochs using the Adam optimizer[7] with a learning rate $10^{-4}$ and a batch size 100.

## Supplementary Note 5: Further results from the first experiment with wavefront shaping

This experiment utilizes the wavefront shaping technique[11] to generate focal spots at the distal end of the fiber. This allows for raster-scanning imaging which can lead to better results due to better SNR ratios. Supplementary Figure 2 (first row) presents speckles from a fixed DMD phase mask recorded at different fiber configurations. The speckle at the calibrated configuration $C_{10}$ represents a simple focal point (Supplementary Figure 2, **(A)**). By bending the fiber away from the calibration position, the focal points become speckles due to coupling between spatial modes of the fiber (Supplementary Figure 2, **(B)** and **(C)**). Supplementary Figure 3 shows the reconstruction quality of GMVAE and AE on APD measurements collected at $C_{10}$, $C_2$ and $C_0$. Both GMVAE and AE give good reconstruction quality on measurements recorded at $C_{10}$. This is expected since $C_{10}$ is the calibrated configuration and was used during training, hence the shapes of the images can be seen directly in the measurements. However, the results show a superior performance for the GMVAE in comparison to AE for the new configurations $C_2$ and $C_0$ which are far away from the calibration position. We can see from Supplementary Figure 4, **(A)** that C-AE could achieve 90% classification accuracy with 86% for GMVAE at the calibrated configuration $C_{10}$. However, GMVAE scores 77% at $C_2$ and 70% at $C_0$ with 7% and 8% enhancement from C-AE, respectively. Supplementary Figure 5 presents the raw data and

latent vectors of 8000 numerical measurements computed at the configurations $C_{10}$, $C_2$ and $C_0$ and projected using principal component analysis (PCA) to the 3D space. In contrast to the raw data (Supplementary Figure 5, left), the GMVAE latent space (Supplementary Figure 5, right) of the testing data points shows a clear separation between the different classes.

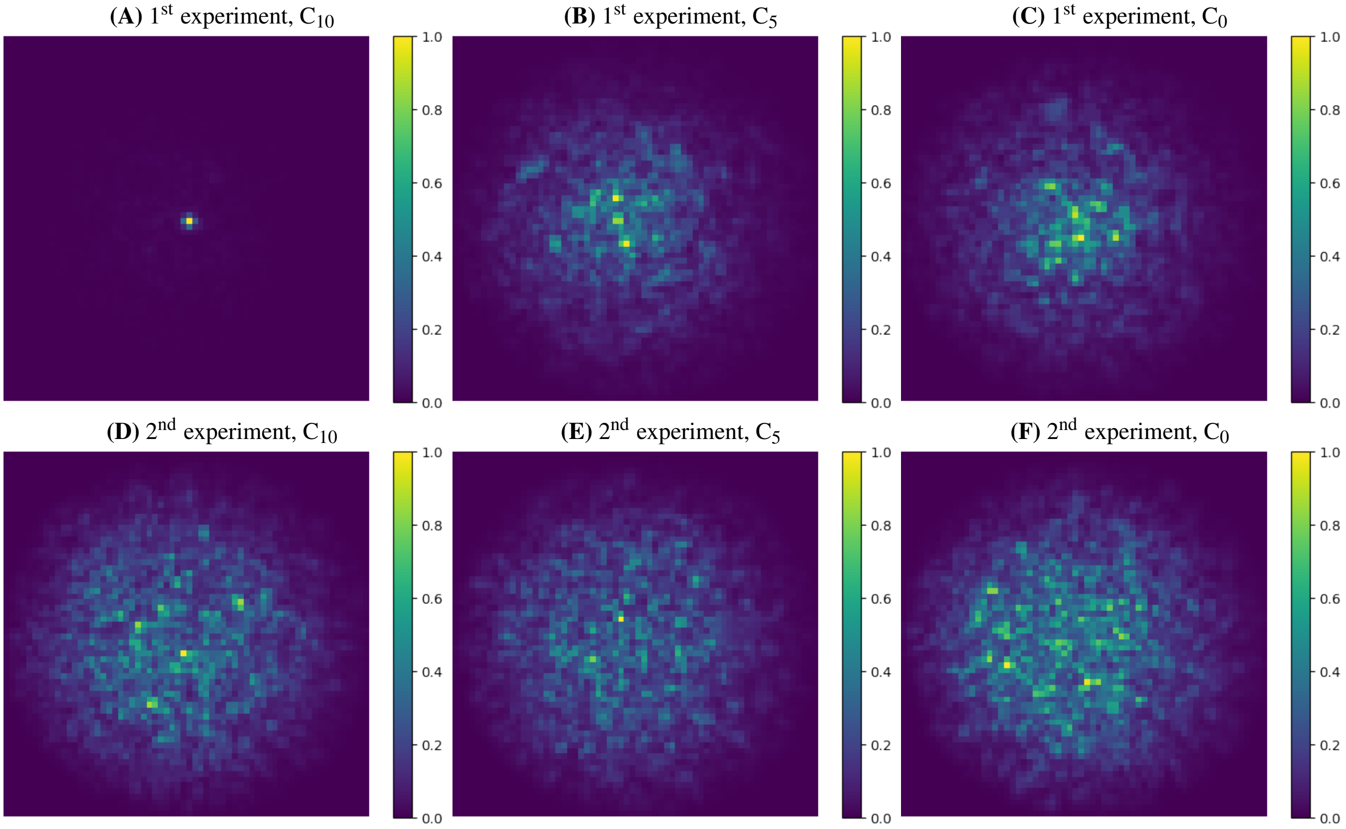


**Supplementary Figure 2.** Speckles from a fixed DMD phase mask recorded at different fiber configurations: $C_{10}$, $C_5$, and $C_0$. **(A)**, **(B)**, and **(C)** show speckles from the first experiment with wavefront shaping, while **(D)**, **(E)**, and **(F)** depict speckles from the second experiment without wavefront shaping.

## Supplementary Note 6: Further results from the second experiment without wavefront shaping

In this experiment, speckles were recorded at the different configurations with no configuration-specific wavefront shaping performed. Supplementary Figure 2 (second row) shows speckles from a fixed DMD phase mask recorded at different fiber configurations. We can see that all configurations are equally challenging. For qualitative assessment, Supplementary Figure 6 compares the reconstruction quality of GMVAE against AE on APD measurements collected at the following configurations: $C_5$, $C_2$ and $C_0$. GMVAE maintained better reconstruction quality compared to AE for all configurations. However, GMVAE overall reconstruction quality was reduced in comparison with the first experiment with wavefront shaping. This is expected since speckles in the first experiment had a higher signal-to-noise ratio (SNR) than speckles in this experiment because most of the energy was concentrated around the focal points leading to a more orthogonal measurement basis. The confusion matrices obtained by GMVAE and C-AE at the configurations $C_5$, $C_2$ and $C_0$ on 8000 numerical measurements from the 8 trained-on classes are shown in Supplementary Figure 7. Once again, the results suggest better classification accuracy for GMVAE compared to C-AE. More precisely, GMVAE scores 79% at $C_5$, 75% at $C_2$ and 66% at $C_0$ with 1%, 7% and 17% enhancement from C-AE, respectively. Finally, the raw data and the GMVAE latent vectors of 8000 numerical measurements computed at the configurations $C_5$, $C_2$ and $C_0$ and projected by PCA to the 3D space are presented in Supplementary Figure 8.

## Supplementary Movie 1: Video reconstruction of static and moving objects during fiber bend alteration

The video, provided as supplementary material, presents the reconstruction of three static objects and one moving object as the fiber is bent from the calibrated position $C_{10}$ (10 mm, 230°) to $C_5$ (5 mm, 255°). This change corresponds to a fiber bend alteration of 25° and a movement range of 4 cm.

## References


1. Jang, E., Gu, S. & Poole, B. Categorical reparameterization with gumbel-softmax. *arXiv preprint arXiv:1611.01144* (2016).
2. Maddison, C., Mnih, A. & Teh, Y. The concrete distribution: A continuous relaxation of discrete random variables. In *ICLR* (2017).
3. Figueroa, J. A. Semi-supervised learning using deep generative models and auxiliary tasks. In *NeurIPS* (2019).
4. Collier, M. & Urdiales, H. Scalable deep unsupervised clustering with concrete gmvaes. *arXiv preprint arXiv:1909.08994* (2019).
5. Weinberger, K. Q., Blitzer, J. & Saul, L. K. Distance metric learning for large margin nearest neighbor classification. In *Adv. Neural Inf. Process. Syst.*, 1473–1480 (2006).
6. Schroff, F., Kalenichenko, D. & Philbin, J. Facenet: A unified embedding for face recognition and clustering. In *IEEE CVPR*, 815–823 (2015).
7. Kingma, D. P. & Ba, J. L. Adam: A method for stochastic gradient descent. In *ICLR*, 1–15 (2015).
8. Li, Y., Xue, Y. & Tian, L. Deep speckle correlation: a deep learning approach toward scalable imaging through scattering media. *Optica* **5**, 1181–1190 (2018).
9. Li, Y., Cheng, S., Xue, Y. & Tian, L. Displacement-agnostic coherent imaging through scatter with an interpretable deep neural network. *Opt. Express* **29**, 2244–2257 (2021).
10. Resisi, S., Popoff, S. M. & Bromberg, Y. Image transmission through a dynamically perturbed multimode fiber by deep learning. *Laser & Photonics Rev.* **15**, 2000553 (2021).
11. Stellinga, D. *et al.* Time-of-flight 3d imaging through multimode optical fibers. *Science* **374**, 1395–1399 (2021).

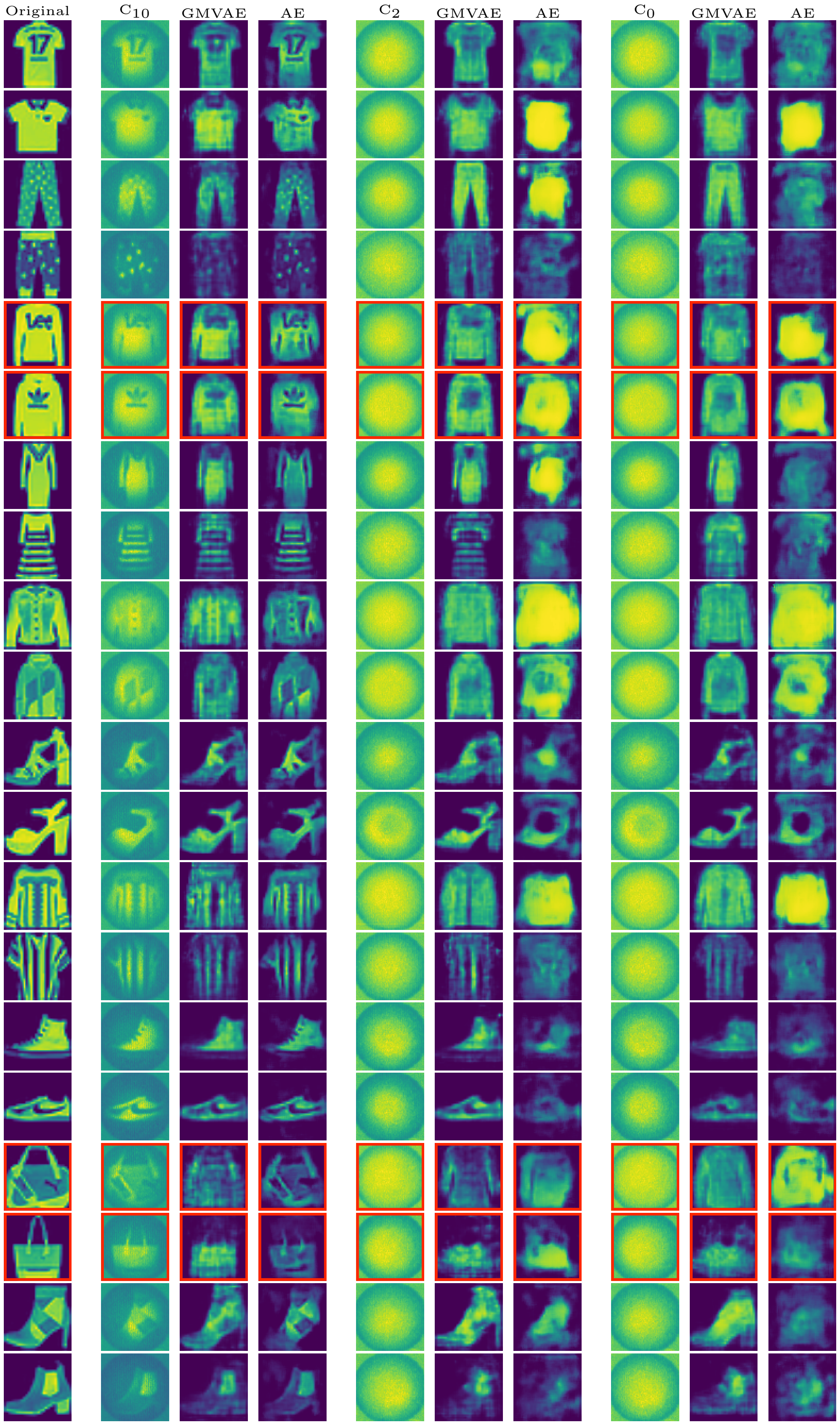


**Supplementary Figure 3.** First experiment with wavefront shaping: reconstruction results at the configurations $C_{10}$, $C_2$ and $C_0$. The first column displays the original images, while each set of three consecutive columns presents the APD measurements, GMVAE reconstruction, and AE reconstruction. Images highlighted by red squares correspond to new classes not used during training. The intensity values of all images in the figure range between 0 and 1.

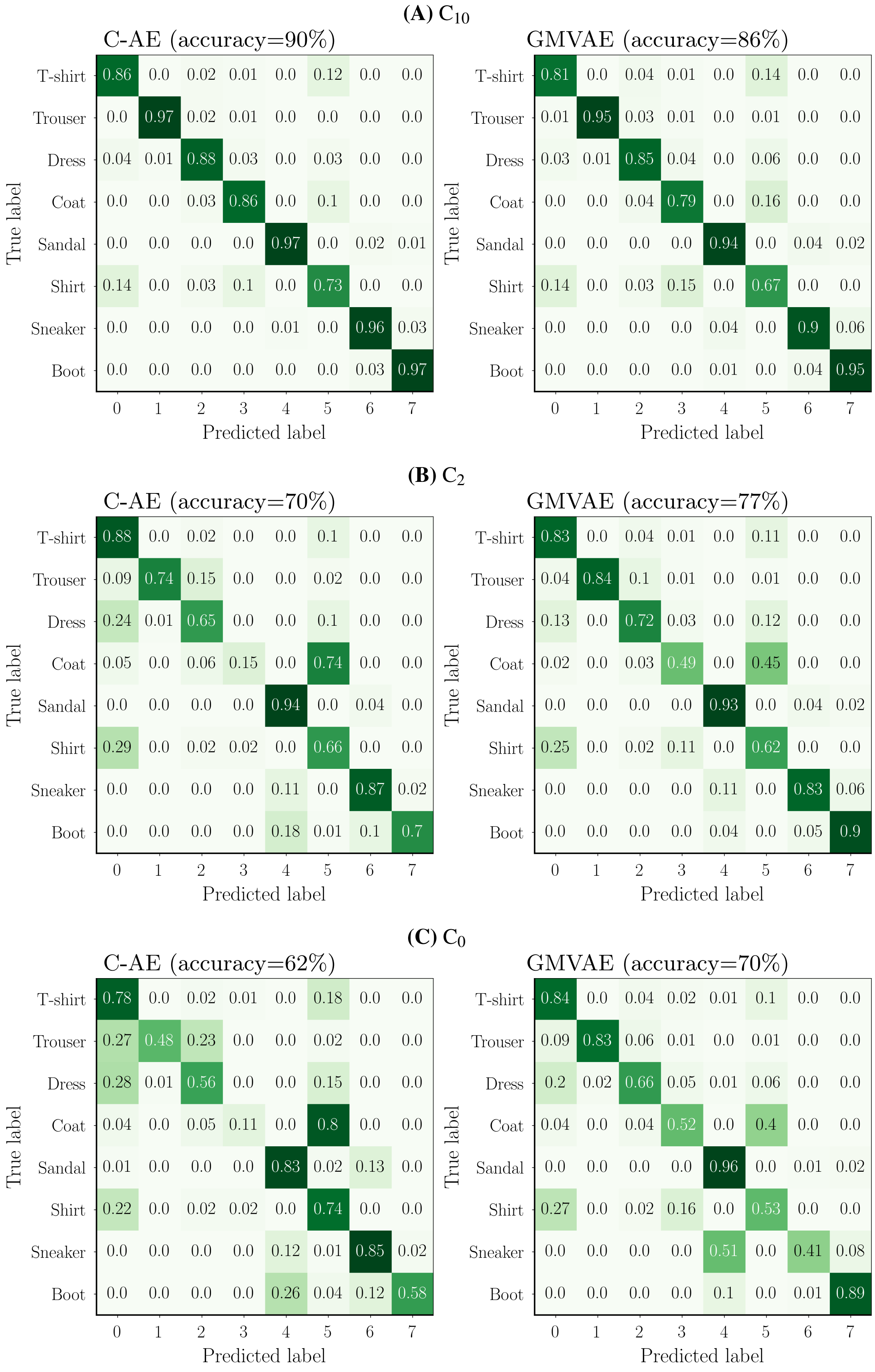


**Supplementary Figure 4.** First experiment with wavefront shaping: confusion matrices obtained by C-AE (left) and GMVAE (right) at different configurations **(A)** $C_{10}$, **(B)** $C_2$ and **(C)** $C_0$ for 8000 numerical measurements from the 8 trained-on classes.

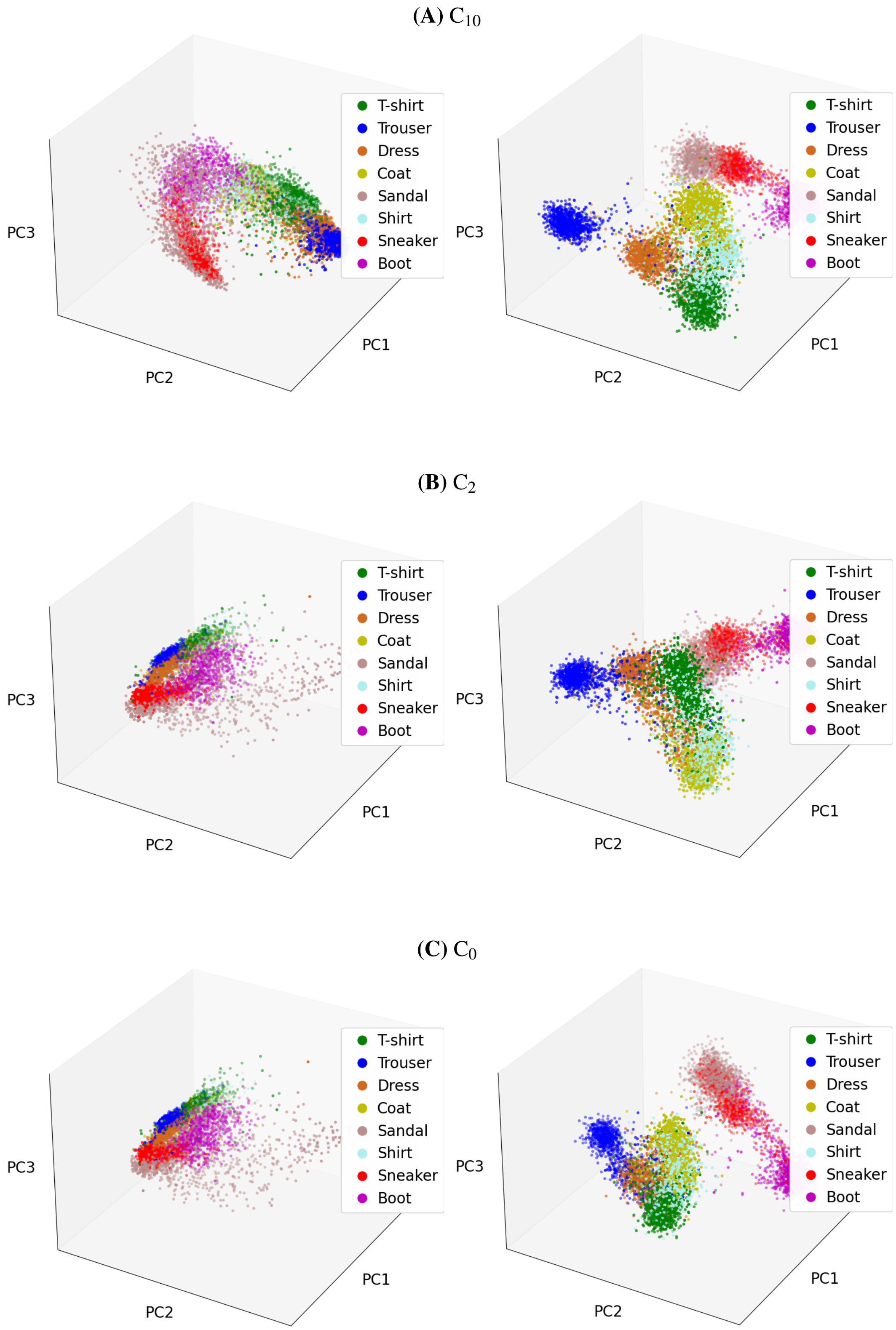


**Supplementary Figure 5.** First experiment with wavefront shaping: 3D PCA projection of the raw (left) and GMVAE latent space (right) vectors obtained at different configurations **(A)** $C_{10}$, **(B)** $C_2$ and **(C)** $C_0$ using 8000 numerical measurements from the 8 trained-on classes.

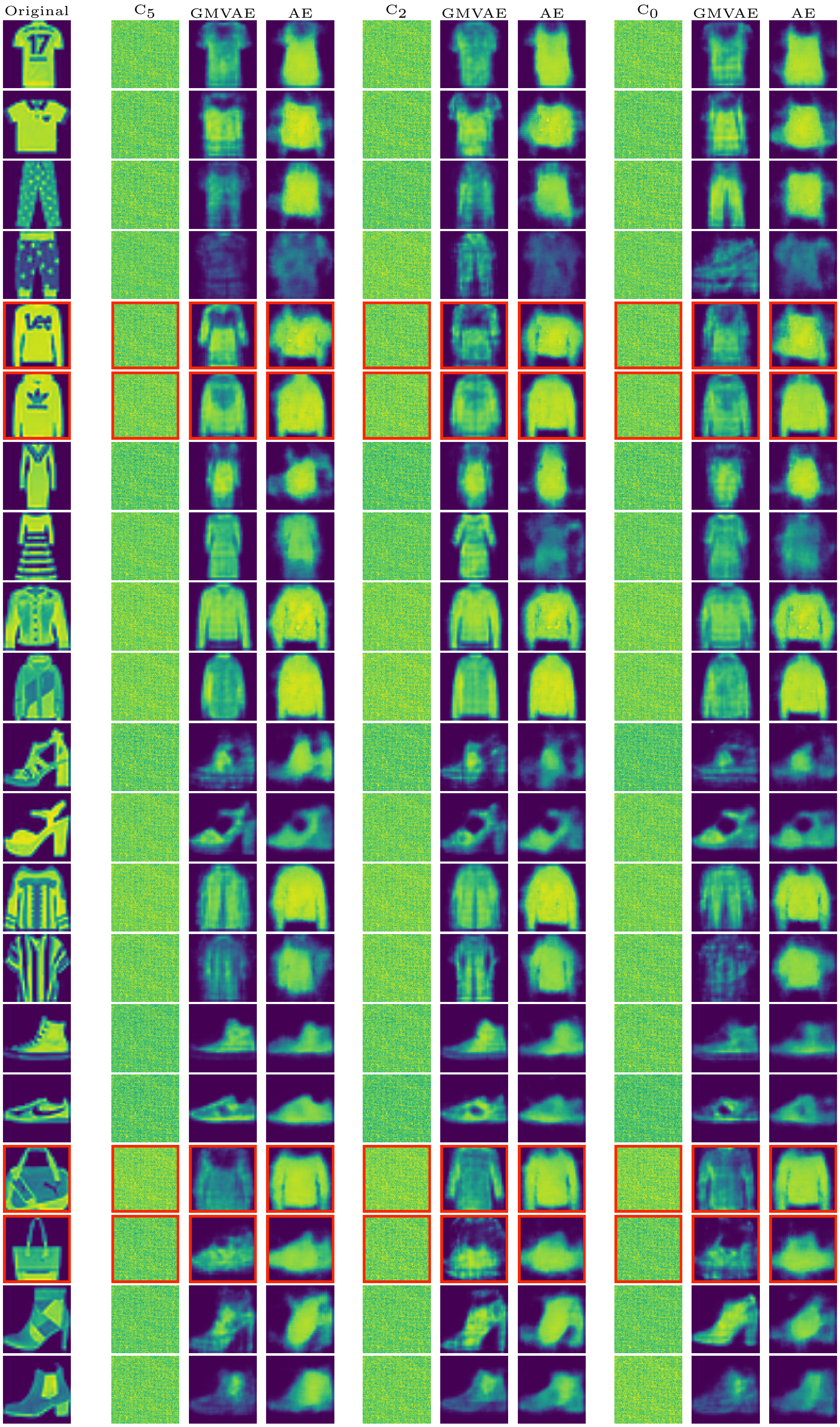


**Supplementary Figure 6.** Second experiment without wavefront shaping: reconstruction results at the configurations $C_5$, $C_2$ and $C_0$. The first column displays the original images, while each set of three consecutive columns presents the APD measurements, GMVAE reconstruction, and AE reconstruction. Images highlighted by red squares correspond to new classes not used during training. The intensity values of all images in the figure range between 0 and 1.

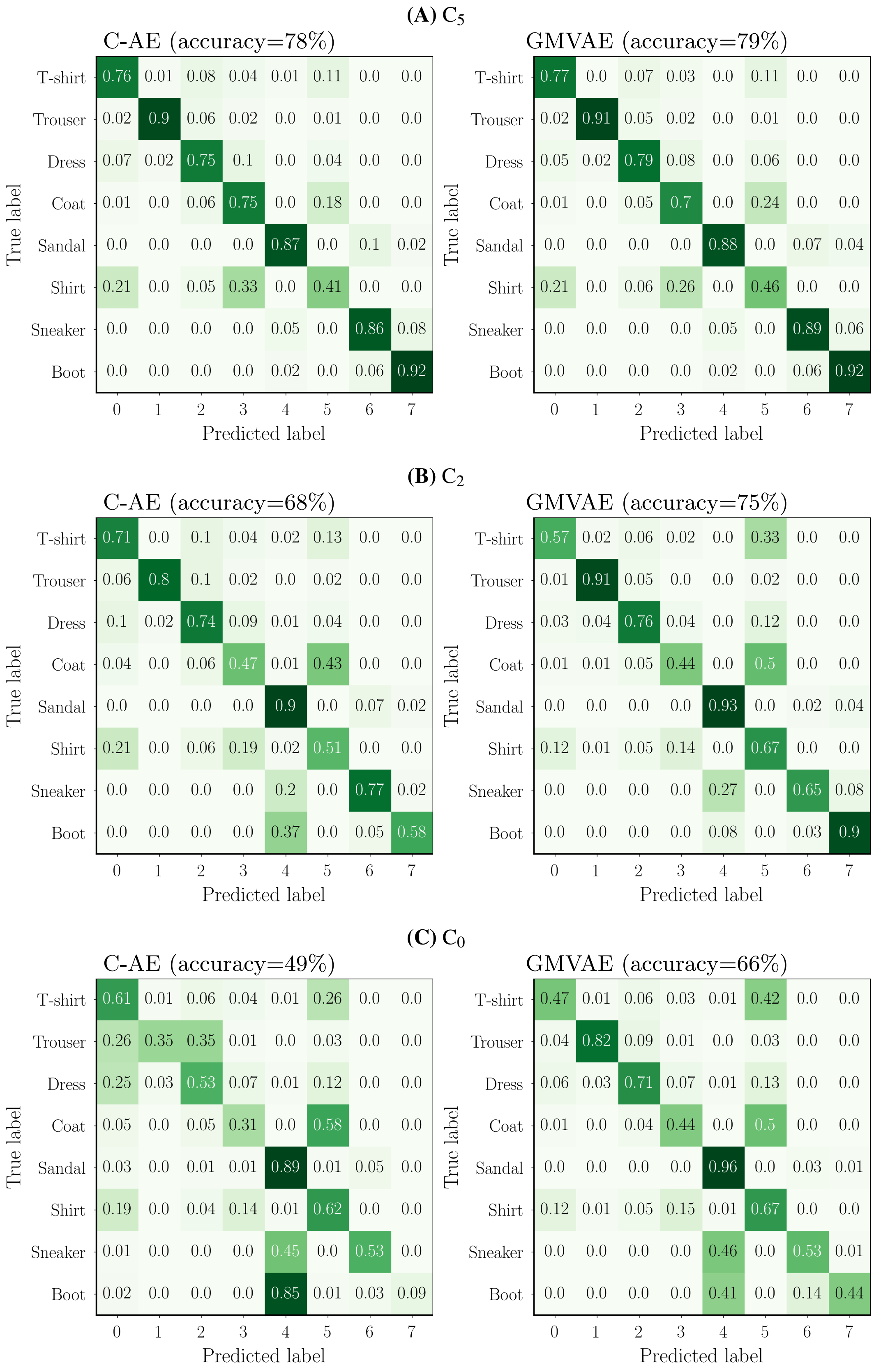


**Supplementary Figure 7.** Second experiment without wavefront shaping: confusion matrices obtained by C-AE (left) and GMVAE (right) at different configurations **(A)** $C_5$, **(B)** $C_2$ and **(C)** $C_0$ for 8000 numerical measurements from the 8 trained-on classes.

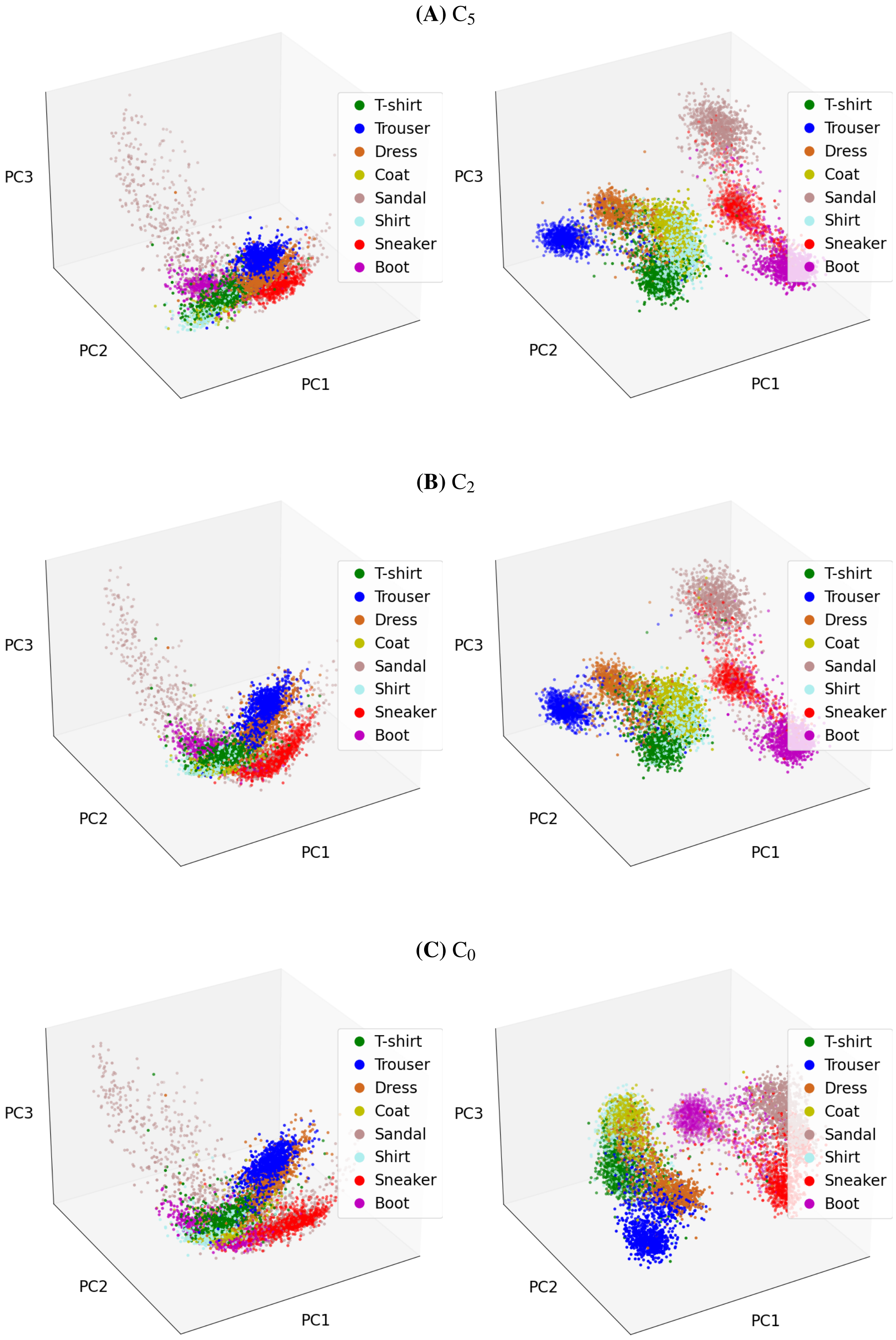


**Supplementary Figure 8.** Second experiment without wavefront shaping: 3D PCA projection of the raw (left) and GMVAE latent space (right) vectors obtained at different configurations **(A)** $C_5$, **(B)** $C_2$ and **(C)** $C_0$ using 8000 numerical measurements from the 8 trained-on classes.
